# Characterizing Surveillance Recommendations From National Comprehensive Cancer Network Guidelines

**DOI:** 10.1001/jamanetworkopen.2025.40727

**Published:** 2025-10-29

**Authors:** Alison S. Baskin, Alina Keshwani, Manvir Bains, Christina Fleischer, Lesly A. Dossett

**Affiliations:** 1 Department of Surgery, University of California, San Francisco; 2 Center for Healthcare Outcomes and Policy, University of Michigan, Ann Arbor; 3 University of Michigan, Ann Arbor; 4 School of Medicine, University of Michigan, Ann Arbor; 5 Department of Surgery, University of Michigan, Ann Arbor

## Abstract

This quality improvement study examines National Comprehensive Cancer Network clinical guidelines for cancer surveillance by type and characterizes specificity and length of recommended treatments.

## Introduction

Cancer surveillance strategies aim to detect recurrence in patients treated with curative intent. Despite a strong theoretical rationale for the benefit of surveillance, empirical evidence does not demonstrate a survival benefit from routine follow-up testing.^1^ Instead, known harms can occur without improvements in mortality or quality of life.^2,3,4^ Despite growing scrutiny of this unfavorable risk-benefit ratio, the scope of surveillance recommendations in the US remains poorly defined. We characterized existing cancer surveillance guidelines to highlight areas of ambiguity, identify potential cases of oversurveillance, and suggest opportunities to improve current recommendations.

## Methods

Between June and July 2025, we systematically reviewed the National Comprehensive Cancer Network (NCCN) Clinical Practice Guidelines in Oncology (NCCN Guidelines) for solid-organ cancers in adults. Two reviewers (A.S.B. and A.K. or M.B.) independently extracted surveillance recommendations following definitive treatment and recorded their NCCN category of evidence. Recommendations were classified by stratification type (eg, risk, stage) and modality (eg, imaging, laboratory testing), and assessed for frequency, duration, and individualization to patient-specific factors. Recommendations addressing only metastatic, recurrent, or progressive disease were excluded. This study followed the Standards for Quality Improvement Reporting Excellence version 2.0 (SQUIRE) reporting guideline for quality improvement studies and was exempt from institutional review board review based on the University of California, San Francisco self-certification protocol.

## Results

Forty-three guidelines provided 483 recommendations across 99 cancer types (Table 1). Four hundred fifty recommendations (93%) of recommendations were assigned category 2A evidence (ie, lower-level evidence with uniform consensus). Although 327 recommendations (68%) were stratified by stage, treatment, or a combination of factors, only 116 (24%) called for individualizing surveillance testing to patient-specific factors (eg, measuring a tumor marker only when initially elevated).

**Table 1.** Frequency and Duration Characteristics of Surveillance Recommendations With Select Examples

Characteristic	Recommendations, No. (%)	Example recommendation (cancer diagnosis and/or region)
**Specify surveillance start^a^**		
Yes	96 (20)	Mammography every 12 mo, beginning 6 mo or more after completion of breast-conserving therapy (invasive breast cancer)
No	387 (80)	Chest CT and CT or MRI of abdomen and pelvis with contrast (unless contraindicated) (pancreatic adenocarcinoma)
**Frequency of surveillance provided^a^**		
Yes	337 (70)	Chest imaging (x ray or CT) every 3 mo (Ewing sarcoma)
No	146 (30)	CBC and chemistry as clinically indicated (squamous cell carcinoma of esophageal and esophagogastric junction cancers)
**Frequency of surveillance provided as a range^b^**		
Yes	215 (64)	PSA every 6-12 mo for 5 y (prostate cancer with initial definitive therapy)
No	122 (36)	Obtain abdomen with or without pelvis CT or MRI annually for 3 y then as clinically indicated. (clinical stage I testicular seminoma)
**Lengthen interval between evaluations over time^b^**		
Yes	216 (64)	CEA every 3-6 mo for 2 y, then every 6 mo for a total of 5 y (stage II-IV rectal cancer)
No	121 (36)	Chest, abdomen, and/or pelvis CT with contrast or chest CT without contrast and abdomen or pelvis MRI with contrast annually for 3 y (stage II-III anal carcinoma)
**Duration of surveillance^a^**		
Clinically indicated or unspecified	234 (48)	CT chest, abdomen, and/or pelvis with oral and IV contrast as clinically indicated (pathologic stage I gastric cancer)
Defined end point	234 (48)	History and physical examination every 3-6 mo for 2 y, then every 6 mo for a total of 5 y (stage II-III colon cancer)
Indefinite or until progression	15 (3)	History and physical examination including complete skin examination every 6-12 mo for the first 5 y, and then at least annually for life (basal cell skin cancer)

Abbreviations: CBC, complete blood count; CEA, carcinoembryonic antigen; CT, computed tomography; MRI, magnetic resonance imaging; PSA, prostate specific antigen.

^a^ Of 483 total recommendations.

^b^ Of 337 total recommendations.

Two hundred twenty-two recommendations (46%) were for imaging, mostly using cross-sectional modalities. Laboratory tests were advised in 109 recommendations (23%), with near-equal distribution across routine (eg, chemistry panel), specialty (eg, hormone panel), and tumor marker laboratory tests. A history and physical or focused clinical examination was recommended in 106 cases (22%), followed by endoscopy in 31 (6%), and pathology testing in 15 cases (3%).

Most recommendations (337 of 483 [70%]) provided guidance on evaluation frequency. Of these, 215 (64%) suggested frequency as a range, and 216 (64%) advised lengthening the interval over time. Regarding duration, 234 (48%) defined an end point and 15 (3%) recommended surveillance testing indefinitely or until cancer progression. An equal number (234 of 483 [48%]) deferred surveillance duration to clinical judgment (ie, “as clinically indicated”) or did not specify further.

## Discussion

In this quality improvement study of current cancer surveillance guidelines, we found that many recommendations were nonspecific and lacked clear guidance on surveillance frequency and duration. As noted in prior studies, this ambiguity leaves significant room for interpretation, likely contributing to variation in clinical practice and the potential for both overmonitoring and undermonitoring.^5,6^

To clarify current surveillance recommendations and improve their utility, we propose several strategies (Table 2). First, recommendations deferring to clinical judgement or offering frequency or duration ranges should include clinical parameters based on the best available evidence to guide intensity of monitoring. Second, to promote individualized care, guidelines should consider patient-specific risk factors and reflect the natural history of the disease (eg, adjusting surveillance intervals based on the expected timing and likelihood of recurrence). Lastly, to enhance the quality and consistency of surveillance guidelines across cancer types, these and other quality improvement strategies could be integrated into a standardized framework outlining the elements necessary for informed decision-making.

**Table 2.** Limitations of Current Cancer Surveillance Recommendations With Suggestions for Improvement

Limitation	Suggestion for improvement
**Quality of supporting evidence**	
All recommendations were supported by NCCN category 2A or 2B evidence, reflecting reliance on low-level data and expert consensus. Guidelines inconsistently cite specific studies or consensus opinions to support individual recommendations	Emphasize high-level evidence: prioritize randomized trials to generate category 1 (higher-level data) on the comparative effectiveness of different surveillance protocols and their benefit compared to clinical history and examination alone or symptom-directed investigations
**Detailed guidance**	
Surveillance frequencies and durations were often provided as ranges or left to the clinician’s discretion (eg, “as clinically indicated”) without further guidance	Provide clinical parameters: when recommendations are flexible, include clinical justifications and examples to guide optimal follow-up evaluation based upon the best available evidence
**Individualization of recommendations**	
Many recommendations did not differentiate surveillance testing strategies based on individual patient risk or disease characteristics	Increased risk stratification and individualization: consider patient-specific factors in surveillance protocols; adjust recommendations based on patient comorbidities, overall life expectancy, and the expected timing, likelihood, and presentation of cancer recurrence
**Completeness and specificity**	
The level of detail across recommendations widely varied, with some guidelines lacking key elements such as surveillance modality, frequency, or end point	Enhance quality and increase consistency: adopt a standardized framework outlining the core elements necessary for informed decision-making across cancer types
**High-risk testing**	
Nearly half of recommendations involved imaging, the majority of which used cross-sectional modalities, despite associated risks of radiation exposure	Consider risk-benefit ratios: regularly reassess the use of higher-risk testing modalities; favor lower-risk options when appropriate; emphasize the importance of shared decision-making when there is increased risk of potential harm

This study is limited by its focus on NCCN Guidelines, which may differ from clinical practice recommendations issued by professional societies (eg, American Society of Clinical Oncology). However, NCCN Guidelines are continuously updated, widely accessible, and developed by multidisciplinary panels, providing broad and up-to-date recommendations that represent the US standard for cancer care decision-making.

## Notes

Supplement. Data Sharing Statement
