# Supplementary material for: Characterizing Surveillance Recommendations From National Comprehensive Cancer Network Guidelines
Source: JAMA Netw Open. 2025 Oct 29;8(10):e2540727. doi: 10.1001/jamanetworkopen.2025.40727 (PMC12573029; doi:10.1001/jamanetworkopen.2025.40727)
Supplement: Supplement. — Data Sharing Statement [file jamanetwopen-e2540727-s001.pdf]

Data Sharing Statement

Baskin. Characterizing Surveillance Recommendations from National Comprehensive Cancer Network Guidelines. *JAMA Netw Open*. Published October 29, 2025.
doi:10.1001/jamanetworkopen.2025.40727

Data
Data available: No

Additional Information
Explanation for why data not available: Data presented in the study are publicly available online through the National Comprehensive Cancer Network (NCCN).
